# Exploring an EAP writing teacher's adaptive expertise and adaptive teaching practices from a CDST perspective

**DOI:** 10.3389/fpsyg.2022.957429

**Published:** 2022-09-29

**Authors:** Xiaoting Xiang, Pengyun Chang, Baohua Yu

**Affiliations:** ^1^Department of English Language Education, The Education University of Hong Kong, Hong Kong, Hong Kong SAR, China; ^2^School of Foreign Languages and Literatures, Chongqing University of Education, Chongqing, China; ^3^School of Foreign Languages and Cultures, Chongqing University, Chongqing, China; ^4^School of Graduate Studies, Lingnan University, Hong Kong, Hong Kong SAR, China

**Keywords:** complex dynamic systems theory (CDST), teachers' adaptive expertise (TAE), adaptive teaching practices (ATP), EAP writing, EFL learners

## Abstract

Teachers' adaptive expertise (TAE) has received increasing attention in the current English as foreign language (EFL) teaching field, however, it has seldom been examined with adaptive practices by teachers in on-going classes among existing literature. Adopting a mixed-method design with data triangulation, this study was conducted to explore the complexity of teachers' adaptive expertise (TAE) and adaptive teaching practices that an EAP writing teacher demonstrated in academic writing courses, from a Complex Dynamic Systems Theory (CDST) perspective. Semi-structured interviews, classroom observations, and questionnaires were arranged to collect qualitative and quantitative data from an EAP writing teacher and 43 EFL learners in a Chinese university. Thematic analysis and SPSS were mainly used in the current work for data analysis. Our findings confirmed (1) the complexity of TAE and ATP with specific features of non-linearity, interconnectedness, and self-organization, which are classic CDST characteristics; (2) the TAE evolved with meta-cognitive, cognitive, affective and social components that are intertwined and contributed to the teacher's adaptive teaching practices (ATP) in her academic writing course; (3) being facilitated by TAE, the teacher's adaptive teaching practices significantly enhanced EFL learners, learning motivation of academic writing and their learning efficiency. Findings of the current work pave the way for future studies in researching TAE and ATP with a thorough consideration of language teachers, students and contexts from the CDST perspective. Moreover, pedagogical contributions are highlighted through the detailed examinations of the EAP writing teacher's ATP, including the class design, teaching plans, and methods, which would be fruitful for the development of tertiary EAP writing research.

## Introduction

Complex Dynamic Systems Theory (CDST) emphasizes the *process* by which *variability and changes* emerge and not on the endpoint of development (de Bot, 2015). The variability that learners demonstrated has attracted the attention of numerous scholars in the field of second language learning (e.g., Zheng and Feng, 2017; Evans and Larsen-Freeman, 2020; Chang and Zhang, 2022). Teachers, as an inseparable and pivotal part of learners' language learning and development, are also been paid close attention to in current L2 research (e.g., Liu et al., 2022). It is affirmed that thinking dynamically about teachers' development, especially their professional progress or regress would shed light on further analysis and understanding of EFL learners' language learning and development (Larsen-Freeman, 2020). Teacher expertise, concerning teachers' superior performance, has attracted consistent researchers' attention (Liu and Chu, 2022). With the shift of studying teacher expertise from cognitive psychology to social and cultural perspective (e.g., Berliner, 2004; Kelly, 2006; Sorensen, 2017), researchers prefer to regard teacher expertise as a dynamic process rather than a stable state (Bereiter and Scardamalia, 1993; Tsui, 2005). In accordance with the CDST and the process view of teacher expertise and adaptive expertise highlights the dynamic and complex process of making flexible adaptations and progressively solving novel issues (Bereiter and Scardamalia, 1993; Bransford et al., 2000), which is essential for teachers concerning the changing work requirements and the ceaseless emergence of new challenges in language teaching (Croskerry, 2018; Lin et al., 2018; Liu et al., 2020; Mees et al., 2020). Moreover, English for Academic Purpose (EAP) teachers in higher education have encountered enormous challenges brought by systematic changes transferring from general English teachers (Li and Morris, 2021). Thus, we aimed to depict the dynamics and complexity of the teachers' adaptive expertise and corresponding adaptive teaching practices in a tertiary-level EAP writing course, and explore the influences exerted on EFL learners' knowledge and understanding of academic writing.

## Theoretical background

### Adaptive expertise

The term adaptive expertise (AE) was first coined by Hatano and Inagaki (1984) in comparison with routine expertise (RE). RE is conceptualized as the capability of performing core skills of routines fast and accurately, while adaptive expertise is more applicable for handling unfamiliar situations or novel problems. Characterized by Holyoak (1991), individuals with adaptive expertise can “invent new procedures derived from their expert knowledge” (p. 310) instead of merely applying already-mastered knowledge and skills, who possess the knowledge of why particular methods or procedures are efficient, under what circumstances, and how to enhance them (Hatano and Inagaki, 1984; Salmon et al., 2020). It is assumed that RE is beneficial to maintaining high-level performance but learning is probably halted (Chi, 2011). However, adaptive experts stay flexible in novel situations and purchase lifelong learning, who “are meta-cognitive and continually question their current levels of expertise and attempt to move beyond them” (Bransford et al., 2000, p. 48). The knowledge structures are enriched and refined with the continuing pursuit of new challenges, which is in line with Bereiter and Scardamalia (1993) idea of progressive problem-solving. Adaptive expertise can thus be understood as a dynamic process of ongoing learning or problem-solving to expand the domain knowledge and skills.

### Teachers' adaptive expertise (TAE)

To understand AE in a more concrete way, researchers have attempted to unveil the specific components of teachers' adaptive expertise (TAE). Crawford et al. (2005) categorized the TAE components into two columns for science teaching: “epistemic and dispositional aspects of adaptiveness”, and “adaptive cognitive and meta-cognitive processes” (p. 7). Consistent with the classification proposed by Crawford et al. (2005), Carbonell et al. (2014) confirmed the differentiation of cognitive and meta-cognitive components through a systematic review. In particular, Crawford et al. (2005) listed relevant cognitive processes (e.g., hypothesis-based reasoning, causal reasoning, and problem-solving) and meta-cognitive procedures including monitoring and assessing the performance and the knowledge states. Carbonell et al. (2014) concluded some other cognitive components, such as cognitive flexibility (the ability to adjust one's reactions in response to environmental changes) and analogical problem-solving (the capability of transferring skills to a context with differences on the surface but similarities on the inside). Carbonell and his group further explained meta-cognitive skills concerning the regulation processes with “self-efficacy, goal-setting and goal achievement, and regulation of emotions” (p. 21). Besides, they highlighted the previous studies reaching consensus on the dispositional components featured with “the openness to experience” (being open to novel issues and desirable for the new experience) or “change-receptivity” (enjoying working in changeable environment contexts) suggested by Griffin and Hesketh (2003). Bell et al. (2012) regarded this type of willingness as the internal motivation to explore innovative methods to better solve problems because individuals with AE do not hold tight to their theories and are eager to break their knowledge limitations (Crawford and Brophy, 2006). Considering the situational complexity, Hatano and Oura (2003) drew researchers' attention to the affective and sociocultural aspects of adaptive expertise, by presenting their observation of how adaptive expertise develops in specific sociocultural contexts with various changes. Taking the same perspective, Baldinger and Munson's (2020) believed that the components of adaptive expertise can be socially constructed by interacting with others and debriefing their teaching experiences and thoughts.

The development of TAE is believed to occur with the enhancement of RE. It is supported by the study of Hatano and Inagaki (1984), which says that TAE and RE share the same knowledge base in familiar situations. The mastery of domain knowledge and skills help teachers display high-level performance with teaching efficiency, accuracy, and fluency (Schwartz et al., 2005; Schoenfeld, 2011). The familiarity with the contexts and accumulated experience enhance the automaticity of instructional actions enabling teachers' quick reactions to the recurring situations (Ropo, 2004), so the teachers' attentions can be spared and riveted on novel problems or teaching attempts. To achieve the optimal adaptability proposed by (Schwartz et al., 2005), balancing efficiency and innovation are the key point. In their model, efficiency at x-axes and innovation at y-axes shape a centered space as the optimal adaptability corridor. It illustrated that routine experts manifest high efficiency but low innovation. Contrarily, the teachers with TAE possess high-level performance at both axes. Therefore, the constant teaching practices seeking high-leveled efficiency and innovation are the feasible way to develop TAE.

### Adaptive teaching practices (ATP)

Some researchers believe that adaptive expertise reveals comparative advantages, especially when accommodating to changes concerning work requirements, environmental complexity, and atypical situations (Chen et al., 2005; Croskerry, 2018; Mees et al., 2020). Therefore, teachers have a particular need for adaptive expertise, who are constantly confronted with unpredictable and variable situations in their teaching work (Lin et al., 2018). Teachers grow to become adaptive teachers depicted by Timperley (2013) as being motivated by a “moral imperative to promote the engagement, learning, and well-being of each of their students” and involved in “ongoing inquiry with the aim of building the knowledge that is the core of professionalism” (p. 5). It is with adaptive expertise that we can gain an insight into what knowledge, skills, and dispositions make teachers immerse themselves in continuous learning via teaching practices to refine their instruction and expertise (Crawford et al., 2005). Yoon et al. (2015) argued that adaptive expertise can be developed through curriculum reforms and adaptive teaching practices. However, scant empirical studies have adopted adaptive expertise as the focal framework to study teachers and their practices (Bowers et al., 2020).

When depicting teachers' practices, scholars tend to utilize the other terms, i.e., teachers' adaptive practices, or adaptive teaching defined by Parsons (2008) as “teacher action that (a) is non-routine, proactive, thoughtful, and improvisational; (b) includes a change in professional knowledge or practice; and (c) is done to meet the needs of a student or an instructional situation” (p. 20). Teachers flexibly adapt their teaching for handling the student diversity and the instantaneous problems “based on continually assessing and learning about students as they teach” (Lampert et al., 2011, p. 1366), who are capable of noticing and interpreting embedded information in students' responses comprehensively and accurately (Gibson and Ross, 2016) and supporting students' differentiated needs (Gallagher et al., 2022). This kind of in-the-moment teaching adaptation conforms to micro-adaptations according to Corno (2008) who also introduces the paired concept, macro-adaptations (changing curriculum and teaching plans for the occurrence of new information). In order to make adaptations, teachers are reflective to assess, monitor, and regulate their teaching practices (Vaughn et al., 2020).

There are two main veins for existing studies concerning adaptive teaching, one is to conceptualize a theoretical model and observe classroom adaptive practices, and the other is to explore ways of developing teachers' adaptive teaching practices. The representatives for the first vein are Parsons and his research team who proposed a cycle model of adaptive teaching encompassing student stimulus, teacher reflection and meta-cognition, and teacher action (Parsons et al., 2018). They have also developed a teaching observation protocol for adaptive literacy instruction (Ankrum et al., 2020). As for the second vein, for instance, Beltramo (2017) analyzed the information from co-generative dialogues to design and enact adaptive classroom practices. Schipper et al. (2018) examined the effects of using lesson study on teachers' self-efficacy and adaptive teaching by quasi-experimental mixed methods. However, the influence of adaptive teaching on students' learning was not fully investigated. One study conducted by Yoon et al. (2019) made the tentative conclusion that there was a positive correlation between TAE and students' understanding and learning of systems.

The above-mentioned review of relevant studies indicates that insufficient explorations have been made regarding the association between TAE and their ATP, and the impacts of adaptive teaching practices on students' learning. In addition, primary and high schools are the main research sites in this field, where tertiary-level institutions are seldom involved.

### Researching EAP writing teachers' adaptive expertise from a CDST perspective

#### Non-linearity development of TAE

Traditionally, linear relationships between cause and effect are generally assumed in language development studies, and so does teachers' expertise. For example, the more considerable time a teacher invested, they would make progress in their teaching methods and expertise; however, there is no direct and linear cause-and-effect relationship from the CDST perspective. Because non-linearity is closely related to the interconnectedness of the system, the more components interact, the more problematic it becomes to predict how the change will occur. In terms of L2 academic writing, teachers' expertise may be influenced by teachers' motivation for teaching academic writing and their own career development, which would further interact with the time they invested in class preparation, as well as their working conditions. Thus, different variables might be interconnected and lead to variations in the TAE. In addition, as for the nonlinear feature of the correlated systems, adaptive teachers tend to manage classes with decentralized control and diversity (Insana, 2015).

#### Complete interconnectedness

All parts within the dynamic system are connected with one another. It has been proved by numbers of empirical studies that connections between systems are equally strong and the mutual impact of changes will be equally effective (de Bot, 2015). TAE is a complex dynamic system that has subsystems such as personal development, interactions between teachers and students, and the teaching environment. Changes in each of them will affect the other parts, accordingly, either stimulating or hindering expertise development of teachers. In other words, when teachers establish interconnections within the class, teachers need to adapt to students' contextual situations, and adaptive teaching facilitates teachers' adaptability (Loughland, 2019). Thus, changes that EAP teachers experienced are assumed to influence their teaching expertise and further affect their EAP writing classes and students.

#### Self-organization (adaptability)

CDST attempts to grasp the nature of the description and explanation of developmental changes. Thus, more recent research attention has been paid to L2 teachers' expertise (Tsui, 2003; Farrell, 2013; Li and Zou, 2017), particularly researching L2 writing teachers' expertise from a CDST perspective (e.g., Yoon et al., 2019; Lee and Yuan, 2021). Studies from the CDST perspective are not the same as the traditional pursuit of an exhaustive taxonomy of factors that might account for any teaching behavior of any given phenomenon. Adopting the CDST perspective, teachers are viewed as a self-organized system whose teaching expertise would adapt with full consideration of the complexity and dynamics of all the ongoing multiple factors of themselves and the contexts, as well as influences from teaching and learning conditions. For example, (Smit et al., 2022) delved into teachers' adaptive teaching through the analysis of the complexity and dynamics of teacher-student interactions in 16 English lessons and confirmed that teachers would automatically alter teaching methods and materials with the ongoing process in the classroom as well as students' responses.

A group of scholars have proposed to adopt CDST in the field of researching L2 teaching and teachers (e.g., Chang and Zhang, 2021; Fogal, 2022), however, fewer empirical studies were conducted, especially in consideration of EAP writing TAE. Therefore, this study will take the stance of CDST to explore an L2 writing TAE and adaptive teaching practices in a tertiary-level EAP writing course. It is hoped that in doing so, the researchers can probably enhance the understanding of EAP writing teachers' expertise development and changes within the complex dynamic teaching process.

## Methods

### Research design

A mixed-method research design was adopted in the current work, with the aim of exploring an EAP writing teacher's adaptive expertise (TAE) and her adaptive teaching practices (ATP) in academic writing courses, moreover, identifying the influences that TAE and ATP generated on university students' understanding of academic writing in terms of academic writing knowledge and genre awareness. A teacher from one first-tier university in China and her students participated in our two-stage study from September 2020 to January 2022. Each year, a 17-week EAP writing course was organized for senior students with English major. Thus, our pilot study was conducted in 2020 (Sep., 2020-Jan., 2021), and our main study was conducted in 2021 (Sep., 2021-Jan., 2022), which are listed in Table 1 with detailed information. Findings of the main study were reported only in the current work because of the word limits.

**Table 1:** Research design of the current study.

**Timeline**	**Participants**	**Major tasks**
**Pilot study**
Sep., 2020	First author and Christine	Semi-structured interview (around 1.5 h)
Oct., 2020- Nov., 2020	Authors, Christine, EFL learners	Classroom observation and informal talks (four weeks)
**Main study**
Sep., 2021	First author and Christine	Semi-structured interview (1 h)
Oct., 2021-Dec., 2021	Authors, Christine, EFL learners	Classroom observations (6 weeks)
Dec., 2021	First author and Christine	Semi-structured interview (1 h)
Sep., 2021 and Dec., 2021	Second author and EFL learners	Questionnaires (two times)
Jan., 2022	First author and EFL learners	Focus group interview (around 2 h)

Specifically, semi-structured interviews were organized two times between the first author and the EAP writing teacher, aiming to unveil her TAE development. Classroom observations were designed to track and record the teacher's adaptive teaching practices in class. EFL learners were invited to complete a questionnaire to reveal their understanding of academic literacy and genre awareness, which were organized at the beginning and end of the teacher's academic course. Focus-group interviews were arranged with five volunteer EFL learners from the academic writing class for intertwining their perceived gains with TAE and ATP. All these quantitative and qualitative data were collected as data source triangulation in the current study to ensure the validity and reliability of our findings.

### Research questions

This study is going to address the following two research questions:

1) How does the teacher's adaptive expertise develop along with her adaptive teaching practices?
2) How do students perceive and gain from the teacher's adaptive teaching practices in EAP writing class?

### Participants

Christine, a female EAP writing teacher, is the major participant in this study. She obtained her Ph.D. degree from abroad and majored in applied linguistics. Currently, she works in a “double first-class” university in China and has been given the EAP writing course for 4 years. Christine is highly motivated in both her career promotion and academic development. Christine was invited to our main study because she had manifested TAE and ATP features in our pilot study and was willing to further participate in the main study.

There were 43 students in Christine's academic writing course in the year 2021. In our main study, all of them cooperated in completing the questionnaire on academic writing knowledge and genre awareness, which were organized at the beginning and end of the course. Altogether, 38 valid questionnaires were collected for data analysis. Among these, five students from the EAP writing course volunteered to participate in our focus-group interview. They are all female, Grade 4, major in English, and 21 years old on average. Anonymous names of Liu, Yang, Wang, Zhou, and Xiaozhi were used in the current study to refer to the five students according to their willingness.

### Instruments

#### Semi-structured interview with Christine

Christine was interviewed by the first author two times with different focuses. The first interview was conducted in September 2021 (the beginning of the current study) to collect information about the course, and to understand the teacher's justifications for the course design. The questions mainly include (1) Can you introduce the course design concerning goals, targeting students, teaching materials, main teaching activities, etc.? and (2) Why do you design it in that way and have you made any adaptations?

The second interview was to find out the teacher's perceptions about her EAP writing teaching after the current study ended, encompassing the following questions: (1) What were your teaching style and main features of your teaching practices? (2) What were the main interactions in class? (3) Had you provided opportunities for students to express themselves and organize their own learning? (4) Was there any part of the teaching catering to the students' diversity? (5) To what degree do you think you had adaptability in teaching this course and why?

#### Focus-group interview with EFL learners

Five learners participated in our (first and the second author) focus-group interview voluntarily. They shared their opinions about similar questions in Christine's second interview, such as “What were the teacher's teaching style and main features in your views?”, “What kind of interactions had you experienced in class?”

#### Classroom observations

Altogether, six EAP writing lessons (90 min each) were observed successively by the first author from October 2021 to December 2021, composing our classroom observation data. A non-participant observation approach was adopted to provide an outsider's perspective with objectivity (Cohen et al., 2017). The focus of the classroom observations lay on the CDST features of adaptive teaching with respect to the class interactions, task diversity, and students' self-organization opportunities. The first author wrote field notes while observing and audio-recording the class.

#### Questionnaire of academic knowledge and genre awareness

A questionnaire was distributed among the 43 EFL students in Christine's academic writing course (17-week), aiming to track the development of learners' changes before and after taking Christine's course. The questionnaire is modified on the basis of Zheng (2021) work and contains two parts of academic knowledge (10 questions) and academic genre awareness (24 questions).

### Data collection and analysis

A semi-structured interview was organized according to our participants' convenience, either on-line or off-line. A group of major questions was asked. The classroom observation audio were listened for several times, and the parts relevant to CDST features were identified and marked. Meanwhile, the documents such as relevant teaching materials and students' writing assignments, and the first author's field notes, were all collected and analyzed for data triangulation. These qualitative data were first transcribed by the first author and then double-checked by the other two authors. Thematic analysis (TA) is adopted with a deductive approach to analyze the qualitative data from our participants' semi-structured interviews, since it is a widely-used qualitative data analysis method, identifying, analyzing, and reporting patterns or themes within data (Braun and Clarke, 2006). To ensure the consistency and accuracy, three authors have engaged in the coding process. Different codes were adopted to illuminate and highlight the dynamic and complex nature of TAE in the academic writing course.

Collected quantitative data from the questionnaire were operated through the paired sample *t-*test by SPSS (version 26.0), aiming to explore the differences that EFL learners might demonstrate in their understanding of academic knowledge and genre awareness before and after the 17-weeks academic writing course.

## Results and discussion

### The dynamic development of TAE and ATP in EAP writing classes

#### Interconnectedness: Decentralized control and multiple interactions

“Being student-oriented” is the key feature that Christine summarized in her EAP writing course, which is quite different from traditional “teacher-centered or teacher-dominant” classes, that is, with decentralized control and multiple interacitons. As she stated, “my academic writing course was mainly composed of interactive activities and discussions”, (Teacher Interview 1) which was “based on students' feedback and communications” (Teacher Interview 2). For example, when “Introduction” was explained, Christine did not lecture about what it looks like, what are the components, or how to compose it, and explained the writing tips like the traditional way of teaching EAP writing. Instead, she provided students with an authentic “Introduction section” from a published paper, invited them to analyze it, and summarized what the Introduction could be like in their views. From the bottom-up process of analysis and discussion, students would bear in mind the genre knowledge of Introduction with Christine's comments and explanation. Christine insisted on scaffolding students' new learning on their previous knowledge and experience and expanding teaching through diverse interactions.

**Figure 1:**
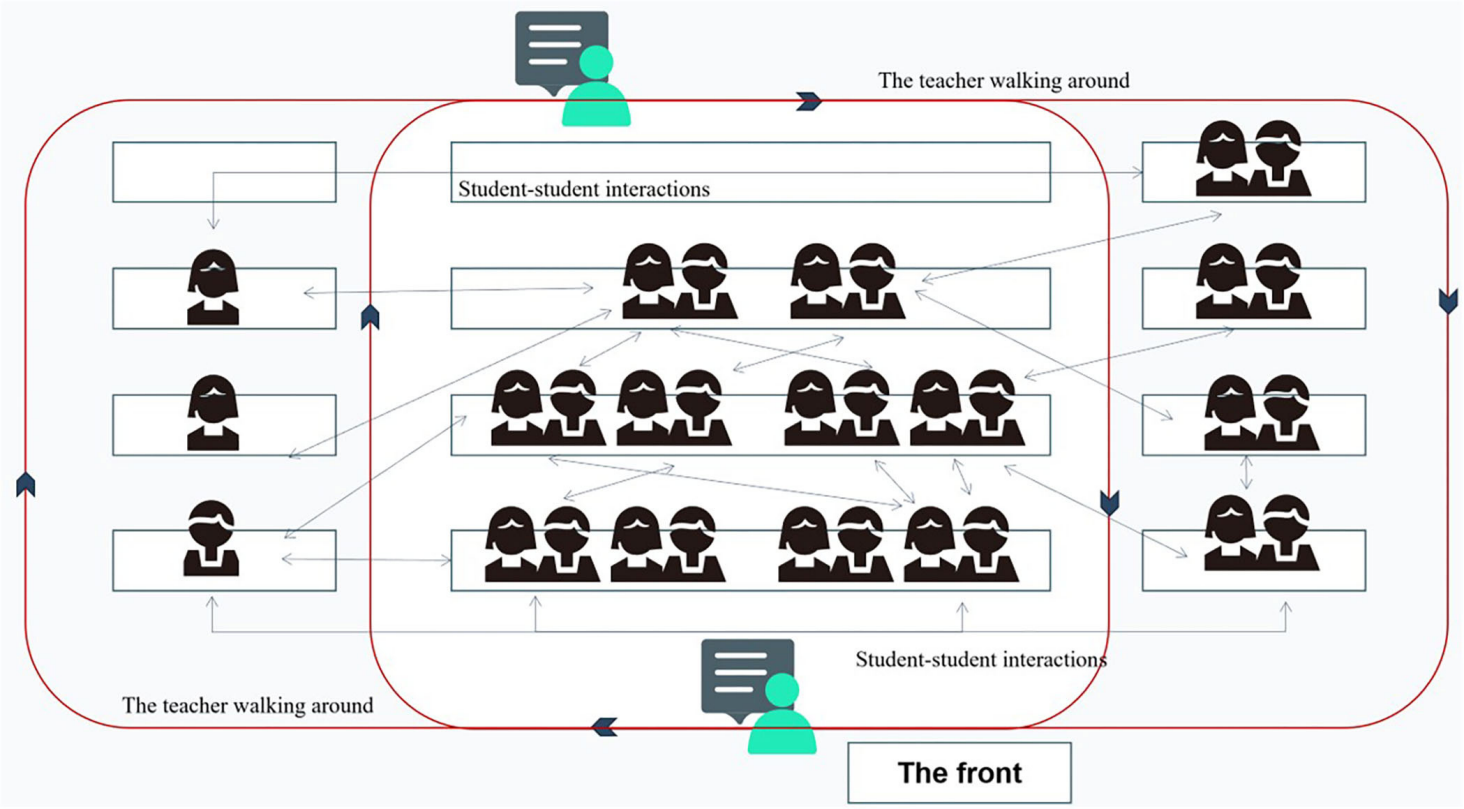
Teacher–student and student–student interactions in Christine's academic writing course.

Figure 1 depicts the trajectories of teacher-student and student-student interactions, indicating Christine's teaching with decentralized control and diversified interactions, which were observed in class.

Figure 1 clearly illustrates that not only were students encouraged to express their ideas in class but also could they share thoughts through multiple student–student interactions. Christine always encouraged her students to physically move in the classroom to approach distinguished opinions from other classmates, rather than murmuring with their desk-mates. Therefore, a student could talk freely to the group members as well as students in other groups. In Christine's opinion, when communicating with different classmates, students could be open-minded, and the physical movement would stimulate passive learning to active learning to some extent. As Christine supposed, if the students encountered partners sharing similar answers, they would feel some back-up and company; If they were in disagreement, they would be stimulated to ponder why and study further. Christine commented, “writing class was laborious, and this kind of sharing could bring students some sense of freshness both physically and mentally, thus making them stay focused” (Teacher Interview 2) She could tell students' involvement and sense of achievement from their facial expressions and attitudes in class. These findings agree with the statement that the teacher and students in the class were interconnected and co-adapted in the complex adaptive classrooms (Insana, 2015).

As for the reasons why Christine managed the decentralized and interactive teaching, it was first because Christine's self-portrait of being a teacher as a friend who was willing to listen, encourage, and equally communicate with the students. Therefore, she intended to create a friendly classroom environment where students were not afraid of expressing themselves and making mistakes. Her intention was derived from the belief that teaching and learning were reciprocal. Second, the relevant learning and teaching experience enabled Christine to think in the students' shoes. She recalled that she had been staying on-campus from her graduation study to working at the university, so she shared similar learning experiences, understood the learning difficulties in EAP writing, and never said anything judgemental like “How can you not know it? How come you are unable to do it as an undergraduate student?” (Teacher Interview 2) Thus, this kind of empathy motivated Christine to adapt her teaching to students' situations. These findings specifically embodied the affective aspect of adaptive expertise emphasized by Hatano and Oura (2003), which has been neglected, however, it is crucial.

Furthermore, interconnections enhanced the teacher's management of the decentralized and changeable class. In other words, Christine's case indicates that the interconnectedness reflected in her teaching practices facilitated the development of her adaptive expertise. To be more specific, first, the consistency of the adaptive teaching style enabled her to become skillful in adaptation. Christine had practiced her EAP writing course for 4 years and insisted on establishing multiple interactions in class, which strengthened her preference for a communicative class environment. As commented, she asserted that “I could control the whole process without rigid dominance of the class and students” (Teacher Interview 2). The second interconnection was reflected in Christine's professional roles. In her view, the maintenance of her adaptability in teaching the EAP writing course was attributed to the research-teaching circle. Christine researched her teaching, involved students as the resource of data, and published papers. As she mentioned, each semester, she collected students' concept maps to evaluate her teaching efficiency and problems to improve. She accordingly adjusted her teaching drawing on the research findings and her academic writing practices. Christine's willingness of doing research urged her to follow the development of the research field, and in turn upgraded her teaching. The connection between being a researcher and a teacher made her capable of implementing interactive and adaptive teaching. To summarize, the consistent practice of adaptive teaching and the correlation between her research and teaching work enhanced the teacher's openness to changes, corresponding to the idea of Griffin and Hesketh (2003) concerning change-receptivity.

#### Non-linearity: Task diversity and individualized learning

It was found that Christine could fluently instruct students with some routines, indicating a smooth process of her teaching in class. First of all, the main teaching content was set with the sessions based on the academic writing process, mainly including literature review, methodology, introduction, results, and discussion. When each part was delivered, she focused on “what-why-how” moves, namely, what it is, why they should write this part, and the how-to strategies with structure and language use as the teaching focus. Moreover, the main teaching procedure of each session was gradually routinized, starting from warm-up, recapping, introducing new learning objectives, students' self-practicing with classmates or individually, teacher checking and feedback, and ending with a class summary. Christine explained that she would not make major adaptations concerning the teaching content and main procedure because she was confident about the appropriateness with the accumulated experience and knowledge of teaching the EAP writing course. As she recalled, “I have taught this course for multiple rounds and I kept writing and publishing, I am sure about the teaching content” (Teacher Interview 1). It is in line with the previous research about teachers' adaptive expertise sharing features of routine expertise as well (Hatano and Inagaki, 1984).

In spite of the routinized arrangement in the class, students were engaged in different practices for each session, such as sample analysis, filling blanks, matching, or sequencing, and changing from individual work, and pair work, to group work. The form as follows elaborates on the diversity of classroom activities being observed in Christine's class and referred to the teaching materials (see Table 2). The teacher explained that “the task diversity could increase the possibility of satisfying the students' individual interests, thus improving their engagement” (Teacher Interview 2). She believed that students thus changed from the state of sitting still, listening, and quietly taking notes, to active thinking and sharing. The class then turned not linear and predictable as the time when the teacher lectured and talked all the time, but changeable and productive with students' active involvement.

**Table 2:** Classroom activities and exercises in Christine's academic writing course.

**AW sections**	**Classroom activities**	**AW exercises**
A. Introduction	1. Sample analysis; 2. Map drawing; 3. Identifying “writing moves”	1. Individual reading work 2. Individual writing work; 3. Pair discussion;
B. Literature review	4. Sample analysis and evaluation; Labeling and statement or matching; Identifying the reporting verbs 5. Stance analysis and discussion	4. Peer review and feedback on writing work; 5. Writing work revision;
C. Methodology	6. Matching the terms and the definitions; 7. Filling tables with classified information; 8. Direction-based excerpts from published papers (reading and analysis)	6. Sentence reorganization (Individual first, then pair check, then group discussion); 7. Group analysis and discussion with direction- based excerpts; 8. Etc.
D. Results	9. Identifying information elements in the provided sample; 10. Completing the sample writing with multiple choice; 11. Categorizing sentences into a full paragraph;	
E. Discussion	12. Results and Discussion excerpts comparison; 13. Language comparison with L.R. in section B (e.g., tense, words); 14. Stance analysis with direction-based excerpts; 15. Identifying commonly used phrases and sentences 16. Etc.	

In addition, when implementing the diverse tasks, Christine deliberately catered to student differences. In general, she paid attention to the students' states being positively engaged or negatively insouciant and made in-the-moment adaptions in class. As she emphasized:

*Since I taught this course each year, I gradually knew although students shared similar features overall, they were distinct individually. My belief of making teaching student-friendly urged me to adapt my teaching to their specific situations. Even when instructing students from the same grade in different classes, my explanations, tones and expressions might vary to suit for the class learning atmosphere and characteristics. (Teacher Interview 2)*

To be more specific, Christine intentionally arranged her teaching considering the differences among students from distinguished directions of English majors (i.e., literature, linguistics, and translation). She guided students to form groups of four to five students from the same research field. Especially when the learning materials or tasks were direction-specific, the students could discuss and share in groups with similar backgrounds. In this way, Christine believed that both students' learning motivation and working efficiency could be improved due to the classification and specification of students' needs in diverse groups. Besides, the teacher left students personal writing tasks. For example, the teacher asked students to hand in their thesis topics, brought the five most relevant papers into the classroom, summarized theses literature, and wrote the introduction part and the overview of the methodology. These assignments were in accordance with the student's own thesis writing process, by which students gained more practice to enhance their personalized learning for EAP writing. Accordingly, the teacher facilitated students' learning by providing individualized feedback to each group or each student in or out of class. In this way, it required the teacher skillfully responded to students' individualized problems. The autonomy of digesting students' ideas and writing freed up the teacher's mind to deal with novel issues. Christine admitted that the familiarity with students' levels, expectations, attitudes, and difficulties in this course made her skilled at flexibly reacting to students' situations. With the increasing familiarity with teaching content and students, she was confident to manifest her time management skills in class as well. Even if part of the class was handed over to students for discussion and sharing, she could allocate proper time for her instruction and students' work to accomplish all the tasks at the end. Christine's case highlighted the significance of developing the TAE along with routine expertise. The autonomation of routinized teacher actions facilitated her immediate response to the recurring situations in class (Ropo, 2004) and made corresponding micro-adaptions (Corno, 2008).

#### Self-organization: Encouraging students' self-regulated learning

Another feature of Christine's adaptive teaching was that students were provided opportunities for organizing activities of self-learning. In group work, after briefing the task requirements, Christine just walked around and stood by the side observing and listening to the group discussions. She stressed that she intervened in their discussion only when they were diverging from the focus or commended them for their efforts on the right track. If some groups accomplished earlier, she encouraged them to move to other groups for information exchange. In the whole process, students needed to arrange their group work by themselves. Moreover, Christine asserted that as the students were required to accomplish their personalized writing, they freely determined how much effort they were willing to exert. She remembered that some students reviewed their notes, explored more after class, revised the methodology writing several times, and proactively asked for further feedback, but some might not care about their writing quality or problems. This kind of free learning atmosphere, as Christine observed, reflected:

*The students actually enjoyed the active participation and self-organization in the tasks, when they felt like the owner of their learning. They were committed to thinking actively instead of negatively accepting knowledge. (Teacher Interview 2)*

Meanwhile, Christine mentioned that despite the students' self-organization in groups from the same branch of English major, she was sensitive to students' organizational problems and learning difficulties and quickly made relevant adjustments. For example, when preparing the class, she especially supplemented samples about results writing to explicitly explain the differences between the fields of linguistics, literature, and translation, because she noticed the absent-minded state of the students' studying literature and translation in analyzing linguistics-related materials.

In order to ensure the teaching accuracy, Christine was self-motivated to search relevant materials online, read research papers and books, and consulted teachers teaching or researching in English literature and translation because her research expertise was applied linguistics and she felt unfamiliar with the knowledge in these two fields. Along with the adaptive teaching, she organized her learning and renewed her expert knowledge as well. As Crawford et al. (2005) and Timperley (2013) stated, adaptive teachers endeavored to build knowledge with continuous learning. At the same time, students were suggested to prepare sampled papers from their point of view to do self-exploration. However, the majority of in-class practices were designed more relatable to linguistics because of Christine's research expertise and experience, and Christine found that students from linguistics seemed more active. She was distressed that “even if I endeavored to deal with the issue of teaching students from diverse directions, my adaptations were limited” (Teacher Interview 1). She proposed that it would be better if experts from the relevant research fields could directly share their knowledge and experience in academic writing in class or EAP writing courses could be set up disparately for each direction. She suggested this to the department and school multiple times but no responses were received, so she could only make self-adjustment. She asserted “I would not give up communicating with our school. In parallel to this, when the school did not take measures, I would not cease exploring solutions myself” (Teacher Interview 1). Christine's persistence in self-adjustment to refine her teaching manifested the characteristics of adaptive expertise, such as and continuous reflection for assessing, monitoring, and regulating teaching (Vaughn et al., 2020), and progressive and analytical problem-solving (Carbonell et al., 2014).

### Students' perceptions and gains

In the focus-group interview, the five students expressed their perceptions about their learning experience in the EAP writing course. It can be concluded that they believed they reaped a sense of achievement and happiness because of the internalized knowledge and enjoyment of diverse classroom activities and felt inspired by exchanging ideas with peers and capable of self-organization owing to the opportunities of self-exploration and the willingness of devotion in learning. In spite of the perceived gains, students' knowledge about academic writing and genre awareness were significantly improved based on the questionnaire results.

#### Gaining a sense of achievement and happiness

Overall, the five students spoke highly of their experiences in the EAP writing course obtaining a sense of achievement and happiness. First, the students emphasized that the knowledge was systematically and logically arranged, so they could clearly plot each part of academic writing concerning what and how to write (e.g., Zhou, Xiaozhi, and Yang). Yang specified the sense of achievement coming from that “the previous conception about writing was broken and rebuilt”. Second, the students were aware of the interconnections of each part since Christine repeatedly summarized the connections and strengthened their comprehension. As Xiaozhi exemplified, when they completed the literature review part and moved to other sessions, Christine could always find opportunities to relate back to writing literature, showing her mastery of all the teaching content, which repeatedly helped them review the knowledge and thus left a deep impression on them. When they could recall what they had learned even after the course ended, they felt fruitful with a huge improvement in understanding EAP writing. Liu summarized “we seemed to obtain a manual for academic writing, according to which we knew what should and should not be composed, and we were equipped with various tools”.

As for gaining a sense of happiness, they stressed that they felt free to share and express themselves in class (Zhou, Xiaozhi, and Liu). They pointed out that it was because the teacher was “willing to listen to us” (Liu) and “encouraging students to talk without condemning our mistakes” (Zhou), so the students “were not afraid and felt close to the teacher” (Xiaozhi). Yang mentioned that the teacher always listened to the student's answers carefully and provided encouraging feedback without harsh judgment. Therefore, the students were, in turn, actively interacting with the teacher, and willing to participate in classroom activities in this “supportive and encouraging environment” (Xiaozhi). Moreover, students were actively engaged because they enjoyed performing various practices in class. Zhou described them as “interesting like playing games but useful”. As Wang commented, “I was happy because I internalized knowledge and enjoyed the activities”. Students happily depicted their experience in doing those exercises, like ordering, matching the right statements, or analyzing the different samples. They specifically felt enjoyable when they compared their answers in groups. They were especially gratified by idea sharing and peer company. They were not afraid or ashamed of making mistakes because they knew they were not alone. Conversely, they regarded it as an opportunity to deepen their learning and sharpen their memory (e.g., Zhou, Wang, and Liu). In addition to the task diversity, the students took delight in working with partners from the same research direction and receiving the direction-specified learning materials. Zhou was an reprentative case, who stressed her satisfaction with this course:

*I was content throughout the whole process, because I took Christine*'*s applied linguistics course before and was familiar with her teaching style. Moreover, the materials and practices in EAP writing course was closely related to my research direction. I enjoyed all the activities, especially when I could also discuss with classmates from the same research field. (Zhou)*

However, there still existed inactive participation among the students. For example, Wang was from another class, she commented that the students in her class were not that active, but the teacher still endeavored to elicit students' ideas by consistently questioning them. Even though she was not actively answering the teacher's questions, she self-checked the comprehension alongside in class and reviewed notes after class. Wang further explained that she was not that contented in class because her research direction was literature and only three students were working alike. She and the other two students had little to discuss because the learning materials about literature were not adequate and they found it hard to make judgment of their limited knowledge about literature research. With the rushed ending of group work, she felt bored in discussion session and just quietly observed the intense interactions in the linguistics groups. Zhou and Wang presented two contradictory cases explicitly explicating the influence of the connectedness between teaching and students' situations. If the teaching content and activity arrangement were closely related and adapted to students' backgrounds and levels, they would be happily and actively involved and vice versa. Besides, the class environment and the state of group members matter. As Zhou pointed out, “if the classmates stayed quiet and the people you discussed with were reluctant to talk, you would have no intention to share any knowledge with them”. The above findings indicated that students themselves were interconnected and co-adaptive in class as well.

To summarize, the students' comments elaborated that the interconnectedness in class endowed learners pleasant learning environment and productive learning outcomes, which was reflected in the connections between the knowledge and the students, the teacher–student relationship, and student–student interactions. When establishing and strengthening the interconnection in teaching, the teacher's mastery of the content and pedagogical skillfulness functioned in the process (Ropo, 2004). Moreover, the teacher's care for the student and intended implementation of diverse learning tasks in class brought a satisfactory learning experience to students, but it was challenging for the teacher to make such adaptations, especially when the domain knowledge was beyond the expertise of the teacher and the students' states were so divergent leading to a classroom management issue. However, the challenges for teachers' adoption of adaptive teaching have been seldom explored in previous studies.

#### Being inspired and self-regulated

The students expressed their appreciation and gains from exchanging ideas with different students as they could move freely to approach other students not sitting beside them. Zhou exemplified that she got to know three male students with whom she had no contact before by performing the exchanging activity. She noticed their divergent characteristics: one was talkative and outgoing, one was quiet and willing to listen to others, and the other was discreet and always shared opinions after careful thinking. Meanwhile, she learned from their habits of thinking and reaped friendship with them. Liu agreed with Zhou and supplemented that she was impressed by the male student who seemed silent but always demonstrated brilliant thoughts after a silence. Not only did building connections with unfamiliar students, but the students were also truly inspired by peer communication. Liu explained that she learned some textual analytical techniques from other students, such as using language expressions and tenses to make inferences. Yang and Wang emphasized her gains from reviewing others' writing. Yang gained the skills of enhancing text coherence by using logical connections, while Wang harvested the classmates' ways of describing data in the results section of a study.

Moreover, students learned to self-organize their individual or group work with efficiency. Zhou stated that “the teacher always let us think by ourselves after questioning and we were also used to exploring the answers on our own before group sharing”. Similarly, Wang's description showed that their group would share after individual preparation, so “everyone made contributions” (Wang). Yang further explicated that their group work arrangement was efficient because each member possessed the same rate of progress and knowledge base. It thus could be inferred that students were taking responsibility for their own studies through self-exploration. Xiaozhi supplemented her example of being devoted even after class:

*I was discontented with academic wring in the beginning as a result of the previous unhappy research experience. However, when I realized the practicality of Christine's AW course, I spent much more time to enhance my comprehension after class. Especially for the writing assignments, it took me days to accomplish for I kept thinking about the content, and then writing along with repetitious knowledge review. (Xiaozhi)*

There were two reasons causing students' devotion to their EAP writing learning in and out of class. First, it was because of the teacher's cognitive and emotional. Zhou elucidated that the teacher always left students adequate time for solid group discussion and walked around offering conductive suggestions, because she knew them well including their levels and problems. Due to the familiarity, the teacher did not give judgmental feedback, making students “brave to explore and manifest their true selves in front of others in class” (Zhou). Xiaozhi accorded with Zhou, explaining “I liked the teacher walking around with timely advice and feedback”, and especially “felt being cared when receiving individual feedback and willing to take assignments even more seriously”. The second reason was the collective construction of a supportive and encouraging learning environment leading to students' sense of responsibility. With constant practices of self-organization, they gradually sensed the enjoyment of individual thinking and idea exchanging as mentioned above. They realized the responsibility for their learning and the peer influences, so they were “focused on the tasks” (Zhou), and were happy to contribute and learn from each other (Wang and Liu).

#### Academic writing knowledge and genre awareness being improved

Results from the EFL learners' reflection on academic writing knowledge and genre awareness are presented in Table 3. Results indicated that learners achieved a better understanding of academic writing, for the scores of their academic writing knowledge increased from 2.77 to 3.43 on average. Moreover, EFL learners are also equipped with better genre awareness, as the results demonstrated in Table 3, from 2.25 to 3.21 on average. Significant differences are identified by the paired sample *t-*test from EFL learners' ratings on the two aspects, which are all below the significant value of 0.01, indicating that EFL learners achieved a better understanding of EAP writing after taking Christine's academic writing course.

**Table 3:** Differences of EFL learners' pre- and post- academic writing knowledge and genre awareness.

	***N***	**Min**	**Max**	**Mean**	**SD**	***p***
Pre-aw knowledg	38	1.90	3.60	2.77	0.39	0.000**
Post-aw knowledge		2.20	4.49	3.43	0.31	
Pre- genre awareness		1.21	3.04	2.25	0.47	0.000**
Post- genre awareness		2.46	4.96	3.21	0.33	

^**^The difference is significant at the 0.01 level.

In summary, when associating the results of the teacher's interviews with students' perceptions and gains, it is revealed that the complex and dynamic features (interconnectedness, non-linearity, and self-organization) of adaptive teaching were acknowledged by counterparts, resulting in effective teaching and positive learning outcomes. The analysis of these results confirms the innovation of this study, namely, a sufficient exploration on teachers' adaptive expertise (TAE) and adaptive teaching practices (ATP), as well as the follow-up impacts on students' learning. All of these successfully filled the current research gap on TAE and ATP from a CDST perspective. Along with polishing and making teaching adapted to students' situations, the TAE developed in the following ways. The increasing familiarity with EAP writing and the students freed the teacher's mind for exploring ways for novel issues, which corresponds with the previous studies on routine and adaptive expertise (Hatano and Inagaki, 1984; Ropo, 2004). These findings highlight the teacher's familarity and fluency of utilizing relevant knowledge (e.g., content knowledge about EAP writing and pedagogical knowledge) and skills (e.g., writing skills and classroom management skills) owing to the accumulation of related experience (i.e., learning, researching, writing, and publishing, and teaching), which facilitates the improvement of cognitive flexibility (Carbonell et al., 2014). This study also identified the teacher's continuous learning and progressive problem-solving (mainly about the issue of catering students' diversity) as being essential for developing TAE (Bereiter and Scardamalia, 1993; Crawford et al., 2005; Timperley, 2013). However, the distinct and crucial contributions of this study underline more on findings concerning the social and affective side of TAE (Hatano and Oura, 2003), such as building connections of her different professional roles (like being both a researcher and an EAP writing teacher), the commitment as a teacher (e.g., caring about students' emotions and encouraging students' multiple interactions), the persistence for making adaptations, and self-adjustment even being distressed sometimes. Indeed, it confirms Baldinger and Munson's (2020) finding that TAE constructs and even develops in social interactions and specific contexts.

## Conclusion

Teacher expertise has always been an important aspect in the EFL learning field, however, the complexity and dynamics that teachers demonstrate might not be paid enough attention in the existing literature, especially in the field of EAP writing. Adopting the complex dynamic systems theory (CDST), one EAP writing teacher's TAE and her ATP were explored and illustrated in the current work. Both the quantitative and qualitative data depicted the complexity of the EAP writing teacher's TAE and ATP, as well as the influence on EFL learners.

To summarize, the results of the current study specified how one EAP writing teacher's adaptive expertise (TAE) developed along with her adaptive teaching practices (ATP) from the CDST perspective. It can be identified from our results that: (1) The EAP writing teacher's TAE was continuously developed on the basis of her empathy and care for her students, which stimulate and direct her to be adaptive in teaching the EAP writing course. This confirms the affective and social component of TAE; (2) The fluent teaching routines and the cognitive flexibility have have facilitated the teacher to react immediately even if students are diversified individuals, demonstrating the cognitive component of TAE; (3) although there were drawbacks in the actual context, the teacher's self-motivation for continuous learning and self-adjustment would push her to continuously perfect her EAP writing instructions, plans, and materials, illustrating the meta-cognitive component of TAE; (4) the EAP writing teacher's adaptive teaching style was found to be interconnected closely with her multiple professional roles (being a researcher and an EAP writing teacher), enhancing the adaptive disposition and cognitive flexibility in TAE. Thus, all of the current findings highlighted that the EAP writing teacher is a complex and dynamic entity as the CDST proposed. She also manifested specific CDST features in TAE and academic writing courses, depicting a nonlinear and adaptive style in her teaching life, which would keep changing with more fluctuations as long as the course is going.

Our findings also imply that TAE has exerted positive influences on students, namely, EFL learners benefited from the TAE and ATP. Students confirmed they were emotionally fulfilled with a sense of achievement and happiness because they have actively engaged in multiple interactions and diversified classroom activities that were proper for their language levels and interests. Moreover, EFL learners reported that they have upgraded self-regulated learners within a supportive and welcomes learning environment which welcomes free idea-exchanging. Overall, our EFL learners have achieved a more comprehensive understanding of EAP writing knowledge and developed better genre awareness after the academic writing course delivered by Christine. Current findings of this study would provide useful and effective pedagogical implications for practitioners to take a dynamic view in delivering EAP writing courses, and to assist EFL learners' academic writing development on the basis of their own TAE development.

It also needs to emphasize that this study only recruited one EAP writing teacher for thorough exploration and detailed analysis. Any generalization of current findings of this study should be undertaken with caution. Further studies or relevant practitioners would benefit from employing CDST in EAP writing research and other aspects of language learning and teaching. It would be of great interest to include more participants and extends a longer period of investigation within the diverse and complex system of TAE.

## Data availability statement

The original contributions presented in the study are included in the article/supplementary material, further inquiries can be directed to the corresponding author.

## Ethics statement

The studies involving human participants were reviewed and approved by Chongqing University. The patients/participants provided their written informed consent to participate in this study.

## Author contributions

XX conceived of the initial idea, fine-tuned by PC and BY. XX and PC designed the study, collected and analyzed the data, drafted the manuscript, revised, and proofread the manuscript. PC finalized the draft for submission. All authors contributed to the article and approved the submitted version.

## Funding

This study was funded by a research grant from China association of higher education under Grant 22WY0404, the Fundamental Research Funds for the Central Universities of China under Grant 2022CDJSKPT22, and research grants from Chongqing Municipal Education Commission under Grants yyk22216 and 22SKJD008. This research is also supported by the third batch of scientific research platform construction projects of Chongqing University of Education: The scientific research platform on the collaborative two-way development of language research and application.

## Conflict of interest

The authors declare that the research was conducted in the absence of any commercial or financial relationships that could be construed as a potential conflict of interest.

## Publisher's note

All claims expressed in this article are solely those of the authors and do not necessarily represent those of their affiliated organizations, or those of the publisher, the editors and the reviewers. Any product that may be evaluated in this article, or claim that may be made by its manufacturer, is not guaranteed or endorsed by the publisher.
